# Nanoscale Secondary Ion Mass Spectrometry for Quantifying Membrane Fouling: Fouling Layer Structure and Chemical Interaction

**DOI:** 10.1002/advs.74734

**Published:** 2026-03-09

**Authors:** Mengfei Wu, Xinhua Wang, Pin Zhao, Weilong Song, Ming Xie

**Affiliations:** ^1^ Jiangsu Key Laboratory of Anaerobic Biotechnology School of Environment and Ecology Jiangnan University Wuxi P. R. China; ^2^ Jiangsu Collaborative Innovation Center of Technology and Material of Water Treatment Suzhou University of Science and Technology Suzhou P. R. China; ^3^ Department of Chemical Engineering University of Bath Bath UK

**Keywords:** calcium ion, forward osmosis, membrane fouling, nanoscale secondary ion mass spectrometry, organic fouling

## Abstract

Membrane technology has garnered considerable attention for applications in wastewater treatment and resource recovery. Nevertheless, membrane fouling remains a major barrier, yet the lack of high‐resolution non‐destructive characterization techniques limits mechanistic understanding and model validation. Here, nanoscale secondary ion mass spectrometry (Nano SIMS) is introduced as a powerful analytical tool to visualize and quantify the fouling layers on forward osmosis (FO) membranes. Using sodium alginate as a model foulant and Ca^2^^+^ as a representative multivalent cation, Nano SIMS enabled simultaneous mapping of organic matter (^1^ ^2^C^−^, ^1^ ^6^O^−^) and Ca‐associated matter (^4^ ^0^Ca^1^ ^6^O^−^), revealing the formation and structural evolution of Ca^2^^+^–organic networks within the fouling layer. A binarization‐based image analysis method was developed to quantify the polymer volume fraction (*φ_2_*), which increased markedly with Ca^2^^+^ concentration and correlated strongly with flux decline, providing direct experimental support for the application of Flory–Huggins thermodynamic theory to the interpretation of membrane fouling. Application to real landfill leachate further demonstrated that Nano SIMS retains strong ion‐recognition specificity and is capable of resolving fouling structures in complex matrices. This work establishes Nano SIMS as a versatile and robust non‐destructive characterization technique for membrane fouling research, offering new opportunities for mechanistic investigation and model development in water treatment technologies.

## Graphical abstract

Nanoscale secondary ion mass spectrometry (Nano SIMS) is established as a powerful analytical tool to visualize and quantify the membrane fouling layers.

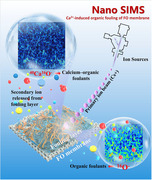

## 1 Introduction

Membrane technology, recognized as one of the most advanced treatment methods, has been extensively applied in water and wastewater treatment over the past decades [1, 2]. However, membrane fouling remains a hindrance for the widespread use of membrane technology because it deteriorates effluent water quality, induces flux decline, and reduces membrane lifespan [3, 4, 5]. Based on the nature of the foulants, membrane fouling is generally classified into three categories: organic fouling, inorganic fouling, and biofouling. Among these, organic fouling is widely regarded as the most challenging to mitigate [6, 7]. Furthermore, multivalent cations—particularly calcium ions—have been shown to aggravate organic fouling by promoting the formation of cross‐linked fouling layers, resulting in more complex and severe fouling states [8, 9]. Forward osmosis (FO) membranes, for example, offer several advantages such as low energy consumption, reduced fouling propensity, and high rejection efficiency in applications like seawater desalination and wastewater treatment [10, 11]. Nevertheless, membrane fouling remains a major obstacle to the stable operation of FO processes [12, 13]. When treating natural water or municipal wastewater containing various organic compounds—such as carbohydrates, proteins, and humic acids—FO membranes are inevitably subject to organic fouling [14, 15, 16]. Furthermore, during the treatment of seawater, reverse osmosis (RO) brines, or landfill leachate with elevated levels of organics and calcium, FO membranes are challenged not only by organic fouling but also by calcium‐associated fouling, including inorganic scaling (e.g., CaCO_3_ and Ca_3_(PO_4_)_2_) and calcium–organic complexes (e.g., calcium alginate) [17, 18, 19]. Therefore, a comprehensive understanding of the mechanisms governing organic fouling—particularly the aggravating role of divalent cations—is critical for the development of effective fouling control strategies.

Non‐destructive analysis of membrane fouling is critical for elucidating fouling mechanisms, and numerous characterization techniques have been developed for this purpose. Confocal laser scanning microscopy (CLSM) has been employed to capture fluorescent images of the microstructure of organic foulants on membrane surfaces [20, 21], while excitation‐emission matrix (EEM) fluorescence spectroscopy enables the identification of dissolved organic matter compositions [22]. Scanning electron microscopy (SEM) [23] and energy‐dispersive X‐ray spectroscopy (EDX) [24] have been employed to observe the deposition morphology and analyze the elemental composition and content within the fouling layer, respectively. Optical coherence tomography (OCT) [25] and micro‐computed tomography (Micro‐CT X‐ray CT) [26] have further advanced the field by enabling non‐invasive visualization and three‐dimensional reconstruction of fouling layer structures. In addition, thermodynamic models such as the Flory‐Huggins theory have been applied to interpret the driving forces behind membrane fouling in filtration processes. Despite these advancements, current analytical techniques for characterizing divalent cation‐enhanced organic fouling remain limited in both resolution and mechanistic insight.

Nanoscale secondary ion mass spectrometry (Nano SIMS), based on the principle of secondary ion emission, offers a novel approach for precise elemental identification and high‐resolution spatial imaging. Nano SIMS has been widely applied across mineralogical and microbial studies. For instance, signals such as ^12^C^14^N^−^ have been associated with fungal biomass and bioactive molecules, while ^56^Fe^16^O^−^ has served as a marker for iron oxides, allowing visualization of mineral–fungi interactions in soils [27, 28]. Similarly, ^12^C^14^N^−^ imaging has enabled the visualization of organic matter morphologies, revealing hollow‐core structures in aerosol particles [29, 30]. Nano SIMS has also been employed to investigate synergistic interactions between fungal roots and minerals [31, 32], microbial utilization of isotopic carbon and nitrogen sources [33], and protein metabolic pathways through isotopic tracing [34]. Membrane fouling characterization has employed various non‐destructive characterization techniques, each with distinct advantages and limitations (details see Text S1 and Table S1). These studies collectively demonstrate the capability of Nano SIMS to distinguish organic matter from elemental ions with exceptional resolution, enabling non‐destructive characterization analyses without disturbing the sample structure. Nevertheless, the application of Nano SIMS for investigating membrane fouling layers has not yet been reported.

In this context, the present study applies Nano SIMS for the first time to analyze the enhancement effect of Ca^2^^+^‐as a representative divalent cation‐on organic membrane fouling. FO was selected as the model membrane process, and sodium alginate (SA) was used as a model organic foulant. Ca^2^^+^ was introduced at concentrations of 0, 100, 200, and 500 mg/L to simulate Ca^2^^+^‐enhanced organic fouling layers. Using a Cs^+^ primary ion source, Nano SIMS enabled detailed acquisition of ion spectra corresponding to organic matter and Ca^2^^+^, facilitating comprehensive analysis of the spatial distribution, structural framework, and accumulation intensity of fouling layers on the FO membrane surface. Furthermore, a binarization algorithm was employed to quantify the organic polymer content within the fouling layer. This quantitative analysis reinforced the applicability of the Flory‐Huggins thermodynamic model, providing deeper mechanistic insights into the development of Ca^2^^+^‐enhanced organic fouling. In addition, a real landfill leachate containing organic matter, calcium, and other foulants was employed as the feed solution in the FO process, and the resulting membrane fouling was characterized using Nano SIMS. This approach not only highlights the robustness of the technique but also underscores potential challenges in data interpretation arising from the coexistence of additional foulants such as silica, sulfate, and barium.

## 2 Material and Methods

### 2.1 Experimental Materials

SA is widely employed as a model organic foulant due to its strong propensity to form an organic fouling layer [35]. To maintain a stable ionic strength, 200 mg of SA was dissolved in 1 L of a buffer solution containing 20 mm NaCl, 20 mm Na_2_SO_4_, and 1 mm NaHCO_3_. Subsequently, calcium chloride (CaCl_2_) was added to the SA solution at concentrations of 0, 100, 200, and 500 mg/L. Both SA and CaCl_2_ were obtained from Sinopharm Chemical Reagent Co., Ltd. (China). The purity of SA was more than 95%, with a molecular weight (Mw) of approximately 200–300 kDa. All solutions were prepared using deionized (DI) water. The resulting solution was used as the FS in the FO filtration system. A 4 m NaCl solution was prepared as the DS. The FO membrane used in this study was composed of cellulose triacetate (CTA) and manufactured by Hydration Technologies Inc. (United States), with an effective membrane area of 0.0034 m^2^.

### 2.2 Experimental Setup

As illustrated in Figure S1, a lab‐scale FO setup was employed for the experiments. The FO membrane was operated with the active layer facing the DS (AL‐DS mode), and spacers were positioned on the DS side to enhance the mechanical support of the membrane and to mitigate reverse salt diffusion during extended operation. During FO operation, the cross‐flow velocity on both sides of the membrane was maintained at 5.92 cm/s. The mass monitoring system consisted of an electronic balance connected to data acquisition software on a computer, which recorded the mass of the FS every five minutes. The software subsequently calculated the average volumetric increment (Δ*V*) over each five‐minute interval. All filtration experiments were conducted in triplicate (n = 3) to ensure reproducibility. All experiments were conducted in a temperature‐controlled room maintained at 25 ± 2°C.

### 2.3 Characterization Methods

Membrane flux is defined as the volume of water transported through a unit membrane area per unit time and is expressed in liters per square meter per hour (L/(m^2^·h), LMH). The conductivity of both the FS and DS was measured at the beginning of the experiment and after 12 h of operation. Ion concentrations were calculated based on the conductivity values, and the osmotic pressure of the solutions was subsequently determined using the following empirical equation [36]:

(1) $$\pi =4.5032C^{2}+43.6426C$$

where *C* represents the ion concentration (mol/L).

The osmotic pressure difference (*Δπ_drive_*) between the DS and the FS provides the driving force for the FO process [37]:

(2) $$\Delta \pi_{\mathrm{drive}}=\Delta \pi_{\mathrm{draw}}-\Delta \pi_{\mathrm{feed}}$$

At the conclusion of each experiment, the FO membrane was retrieved and cut into 5.0 mm × 5.0 mm sections. To preserve the morphology of the organic fouling layer as accurately as possible, the membrane samples were first frozen in liquid nitrogen for 10 min and subsequently freeze‐dried under vacuum at −40°C for 12 h to remove residual moisture. The pretreated membranes were then sputter‐coated with gold and examined using SEM‐EDX (SU8600, Hitachi, Ltd., Japan) to observe the surface morphology and analyze elemental composition [38]. In addition, the distribution of organic fouling on the FO membrane surface was analyzed using CLSM [20]. Given that SA was the sole organic matter in the FS, Calcofluor White (CW), a fluorescent brightener dye for polysaccharides, was employed to stain the organic foulants. 3D image analysis softwares, including PHLIP (Version 0.7), ImageJ, and MATLAB 2007, were utilized to process CLSM image data, enabling quantification of the average amount and thickness of the organic fouling layer. For each experimental condition, at least three membrane samples from independent filtration runs were examined by CLSM and Nano SIMS. The reported thickness values and elemental distributions represent the mean of multiple measurements (n = 3). Further details regarding the application of the Flory‐Huggins theory are provided in Text S2.

### 2.4 Nano SIMS Analysis

The Nano SIMS operates in a simultaneous secondary ion collection mode, where an electron multiplier detects ions via pulse counting, enabling the concurrent acquisition of seven ionic species with high spatial and mass resolution [29, 39, 40]. During SIMS analysis, a Cs^+^ source generates Cs^+^ primary ions with an impact energy of 16 keV for sample sputtering and analysis. The primary ion beam is focused onto the sample, producing high‐resolution digital images of specific elements. To detect electronegative elements, the primary ion beam, with a current of 1–6 pA, is focused to a nominal spot size of 100–200 nm and rastered across the sample surface with a resolution of 256 × 256 pixels. The raster area ranges from 6 to 100 µm^2^, with a dwell time of 1–7 ms per pixel. The intensity of secondary ions is collected over the sputtering period to obtain depth profiles.

In this study, a high‐resolution Nano SIMS (Nano SIMS 50L, Cameca, France) at the Institute of Surface‐Earth System Science, Tianjin University, was employed to visualize the spatial distributions of organic matter and calcium within the fouling layer. The FO membrane samples were mounted onto 10 mm diameter silicon wafers. Prior to Nano SIMS analysis, a 5 nm platinum (Pt) conductive coating was applied to enhance conductivity. The Cs^+^ primary ion beam was used to increase the yield of electronegative elements, sputtering negative secondary ions from a targeted 50 × 50 µm^2^ area, specifically ^1^ ^2^C^−^, ^1^ ^6^O^−^, and ^4^ ^0^Ca^1^ ^6^O^−^. Finally, the Nano SIMS data was analyzed using ImageJ software with the Open MIMS plugin (Harvard, Cambridge) [27, 34, 41]. For Nano SIMS quantification, the analytical uncertainty was estimated at ±5%–10% based on instrument precision and counting statistics.

### 2.5 Experiments with Real Landfill Leachate

To enhance the environmental relevance of the Nano SIMS analyses of membrane fouling, real landfill leachate containing organic matter, calcium, and other foulants was used as the FS in the FO process. The leachate was collected from the Taohuashan Landfill Site (Wuxi, China), and its water quality and elemental composition are provided in Section S2. Since organic foulants complexed with Ca^2^^+^ exhibit minimal fouling layer formation under acidic conditions [42], the leachate pH was adjusted to 4.5 and 7.0 using 0.1 m HCl and 0.1 m NaOH, respectively, to differentiate the distribution of Ca^2^^+^ within the fouling layer. Both pH‐adjusted leachate samples were applied as FS and operated in the FO cell for 12 h, with 4 m NaCl serving as the DS. All experiments were performed in triplicate (n = 3) to ensure reproducibility.

## 3 Results and Discussion

### 3.1 The Performance of FO Membrane

**FIGURE 1:**
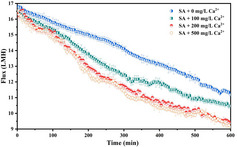
Water flux profile of the FO membrane under different feed solutions.

Figure 1 depicts the variation in water flux over time for different FS containing 200 mg/L SA and varying concentrations of Ca^2^. It was evident that the average rate of flux decline increased from 0.56 LMH/h to 0.71 LMH/h as the Ca^2+^ concentration rose from 0 to 500 mg/L, corresponding to a 29.0 ± 1.2% increase in flux decline. This demonstrates that Ca^2^^+^ exacerbates organic fouling of the FO membrane. This phenomenon is attributed to the ability of Ca^2^^+^ to form coordination bonds with oxygen‐containing groups in SA, thereby reinforcing the organic fouling layer [9, 12, 43]. Interestingly, the flux decay rate at 200 and 500 mg/L Ca^2^^+^ was comparable, at 0.69 and 0.71 LMH/h, respectively. This suggests the existence of a threshold concentration of Ca^2^^+^ for enhancing the organic fouling layer, a phenomenon also observed in previous studies [9, 43].

### 3.2 Non‐destructive Characterization Analysis of Membrane Fouling via Traditional Tools

**FIGURE 2:**
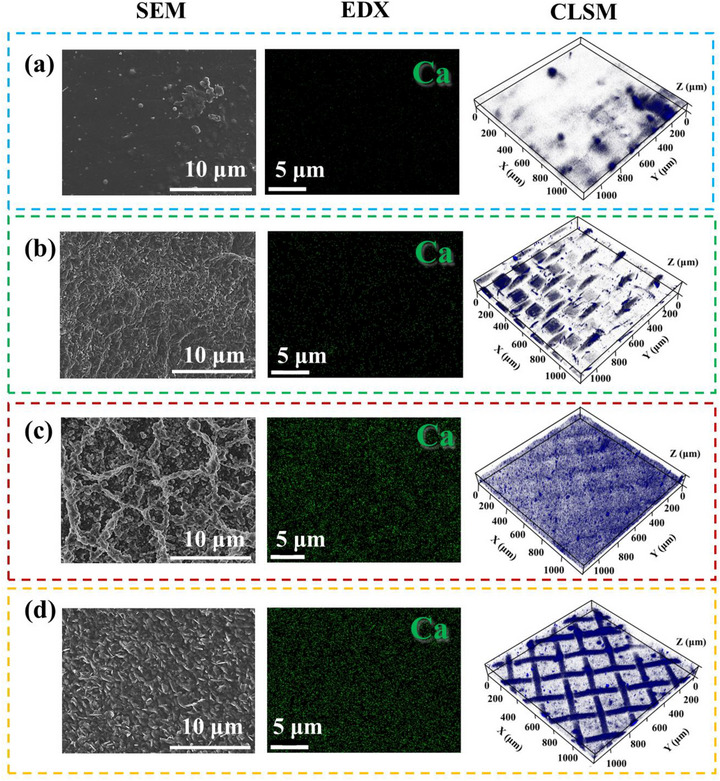
The SEM images, distribution of Ca^2+^ under EDX and CLSM images of the fouled FO membranes at different FS. (a) SA + 0 mg/L Ca^2+^; (b) SA + 100 mg/L Ca^2+^; (c) SA + 200 mg/L Ca^2+^; (d) SA + 500 mg/L Ca^2+^.

SEM and EDX are commonly utilized to visually represent the morphological distribution and elemental composition of organic fouling on membrane surfaces. As shown in Figure 2, the SEM images reveal the surface morphology of the fouling layer formed by varying concentrations of Ca^2^^+^ and SA. Notably, when the Ca^2^^+^ concentration reached 200 mg/L, the organic foulants within the fouling layer formed a distinct net‐like structure, likely due to the chelation and bridging effects of Ca^2^^+^. The EDX images demonstrated an increase in the amount of Ca^2^^+^ detected in the fouling layer as the concentration of Ca^2^^+^ in the FS increased. Detailed quantitative data can be found in Table S2. These findings confirm the accumulation of Ca^2^^+^ on the membrane surface. Previous studies suggest that Ca^2^^+^ interacts with the organic polymer, acting as the “joints” within the structural framework of the fouling layer [44]. However, the SEM images of the fouling layers primarily reveal surface morphology, providing limited detail and monochromatic representations. Similarly, EDX fails to accurately determine the spatial distribution of Ca^2^^+^. These limitations highlight the inadequacy of conventional analytical tools in intuitively explaining the phenomenon of Ca^2^^+^‐enhanced organic fouling.

CLSM has been extensively employed as a crucial method for non‐destructive characterization and observation of organic fouling during membrane processes [21, 38, 45]. In this study, CLSM was used to analyze the fouling layers, enabling the assessment of SA content and distribution. Notably, the CLSM images show pronounced macroscale heterogeneity in the fouling layer distribution across the membrane surface. These patterns are reflected by regions with varying fluorescence intensities, indicating non‐uniform deposition of foulants. As shown in Figure 2, increasing the Ca^2^^+^ concentration resulted in both a higher deposition of SA on the membrane surface and a thicker fouling layer. This observation aligns with the results presented in Table S3, further confirming that higher concentrations of Ca^2^^+^ exacerbate organic fouling on the membrane surface. While CLSM is an excellent tool for quantifying the amount of organic foulants on membrane surfaces, it can not provide non‐destructive characterization measurement of Ca^2^^+^. Therefore, more advanced techniques and methodologies are necessary to explore the underlying mechanisms of Ca^2^^+^‐enhanced organic fouling.

### 3.3 Non‐destructive Characterization Analysis of Membrane Fouling Using Nano SIMS

**FIGURE 3:**
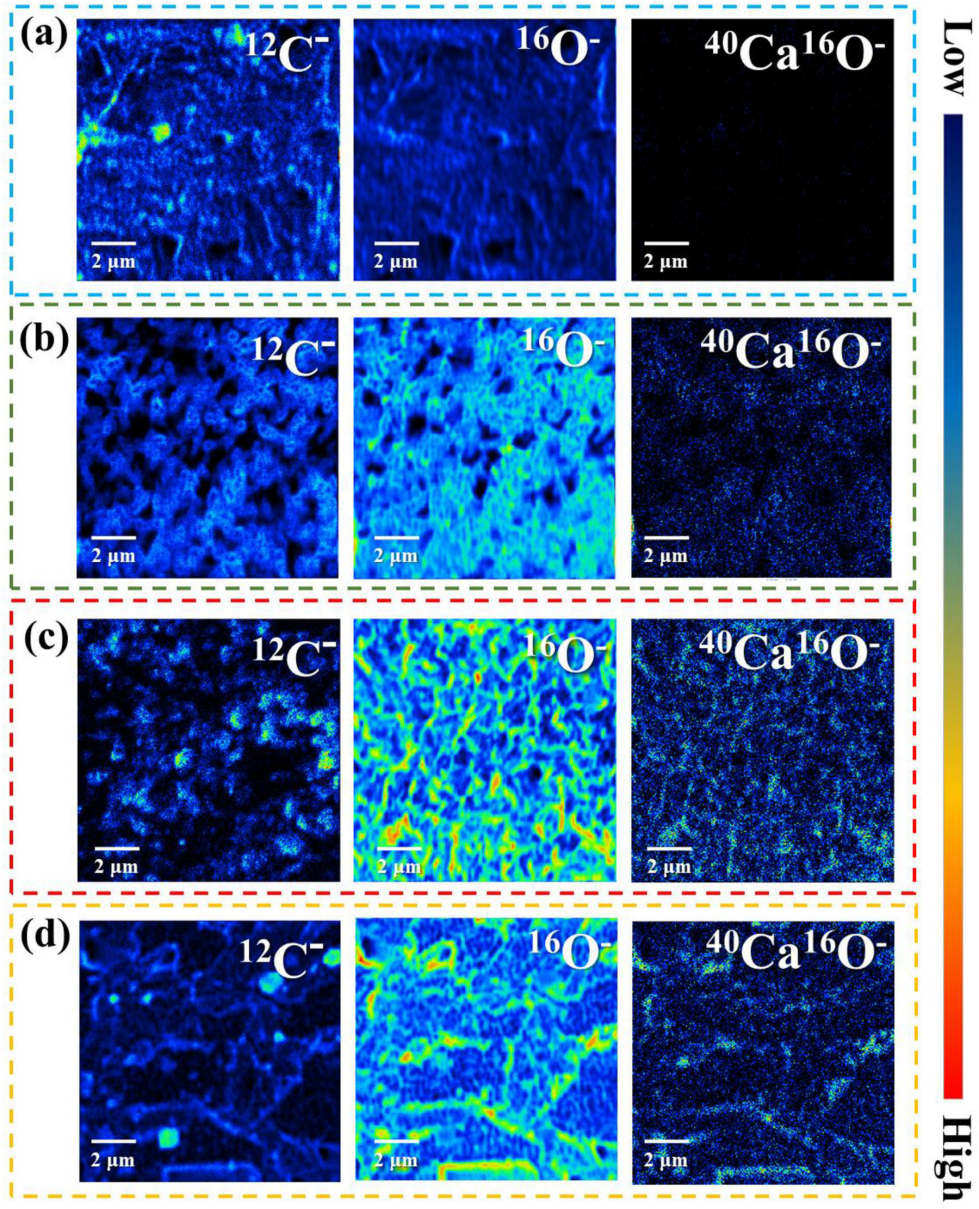
Nano SIMS images of the fouled FO membranes at different FS. (a) SA + 0 mg/L Ca^2+^; (b) SA + 100 mg/L Ca^2+^; (c) SA + 200 mg/L Ca^2+^; (d) SA + 500 mg/L Ca^2+^.

Figure 3 presents the Nano SIMS ion spectra of the fouling layer on FO membranes at different Ca^2^^+^ concentrations. The Nano SIMS image data was processed to illustrate variations in elemental content. As shown in Figure 3, the intensity of ^1^ ^2^C^−^ and ^1^ ^6^O^−^ increased with the rise in Ca^2^^+^ concentration. Both ^1^ ^2^C^−^ and ^1^ ^6^O^−^ are commonly used as indicators of organic matter [29, 34, 41, 46], suggesting that higher Ca^2^^+^ concentrations lead to increased deposition of organic foulants on the membrane surface. This observation corroborates the results from CLSM (Figure 2) and confirms that Ca^2^^+^ enhances the formation of the organic fouling layer. Moreover, the Nano SIMS images revealed distinct differences in the spatial distribution of organic matter. At Ca^2^^+^ concentrations of 0 and 100 mg/L, the network structure of organic matter appeared relatively unclear, while a well‐defined network‐like structure emerged in the fouling layer at Ca^2^^+^ concentrations of 200 and 500 mg/L. This structural transition aligns with the SEM observations in Figure 2. This phenomenon can be mechanistically interpreted through a hierarchical, multi‐scale process: (1) at the molecular level, Ca^2^^+^ forms ionic cross‐links with the carboxyl groups of SA via the “egg‐box” binding mechanism, generating localized gel networks; (2) these cross‐linked segments subsequently aggregate and undergo phase separation during deposition, forming densified gel clusters; and (3) the continued accumulation of these gel aggregates during filtration gives rise to the microscale network structures observed by Nano SIMS. Importantly, increasing Ca^2^^+^ concentration enhances both the degree of molecular cross‐linking and the compaction of the gel network, resulting in a denser and more hydraulically resistant fouling layer. This structural densification directly accounts for the accelerated flux decline shown in Figure 1, as the compacted gel network reduces the porosity and water permeability of the fouling layer.

Beyond conventional methods, Nano SIMS also provides the ability to characterize the spatial distribution and content of polyvalent cations, such as Ca^2^^+^. Nano SIMS tracks Ca^2^^+^ bonded with oxygen via a Cs^+^ ion source, producing images of ^4^ ^0^Ca^1^ ^6^O^−^ (Figure 3). The absence of detectable Ca^2^^+^ is evident in Figure 3a, while Figure 3b–d clearly demonstrates the distribution of Ca^2^^+^. Furthermore, the network‐like structure of Ca^2^^+^ distribution within the organic fouling layer becomes more pronounced at 200 and 500 mg/L Ca^2^^+^, indicating that Nano SIMS successfully visualizes the spatial distribution of Ca^2^^+^ bonded with SA in the fouling layer. Notably, at a concentration of 200 mg/L Ca^2^^+^, the ^4^ ^0^Ca^1^ ^6^O^−^ distribution within the fouling layer appeared most uniform and compact. In contrast, at 500 mg/L Ca^2^^+^, the images displayed larger pores in the network, suggesting a less dense fouling layer. This finding further supports the conclusion that the 200 mg/L Ca^2^^+^ concentration exerts the strongest complex‐strengthening effect with the organic foulants (200 mg/L SA). A lower concentration of Ca^2^^+^ (100 mg/L) results in a weakly bonded and less dense organic layer, while the fouling layer formed at 500 mg/L Ca^2^^+^ is also less dense. These results align with the trends observed in Figures 1 and 2. Overall, Nano SIMS provided precise and detailed insights into the non‐destructive characterization of the spatial distribution of Ca^2^^+^ within the organic fouling layer.

**FIGURE 4:**
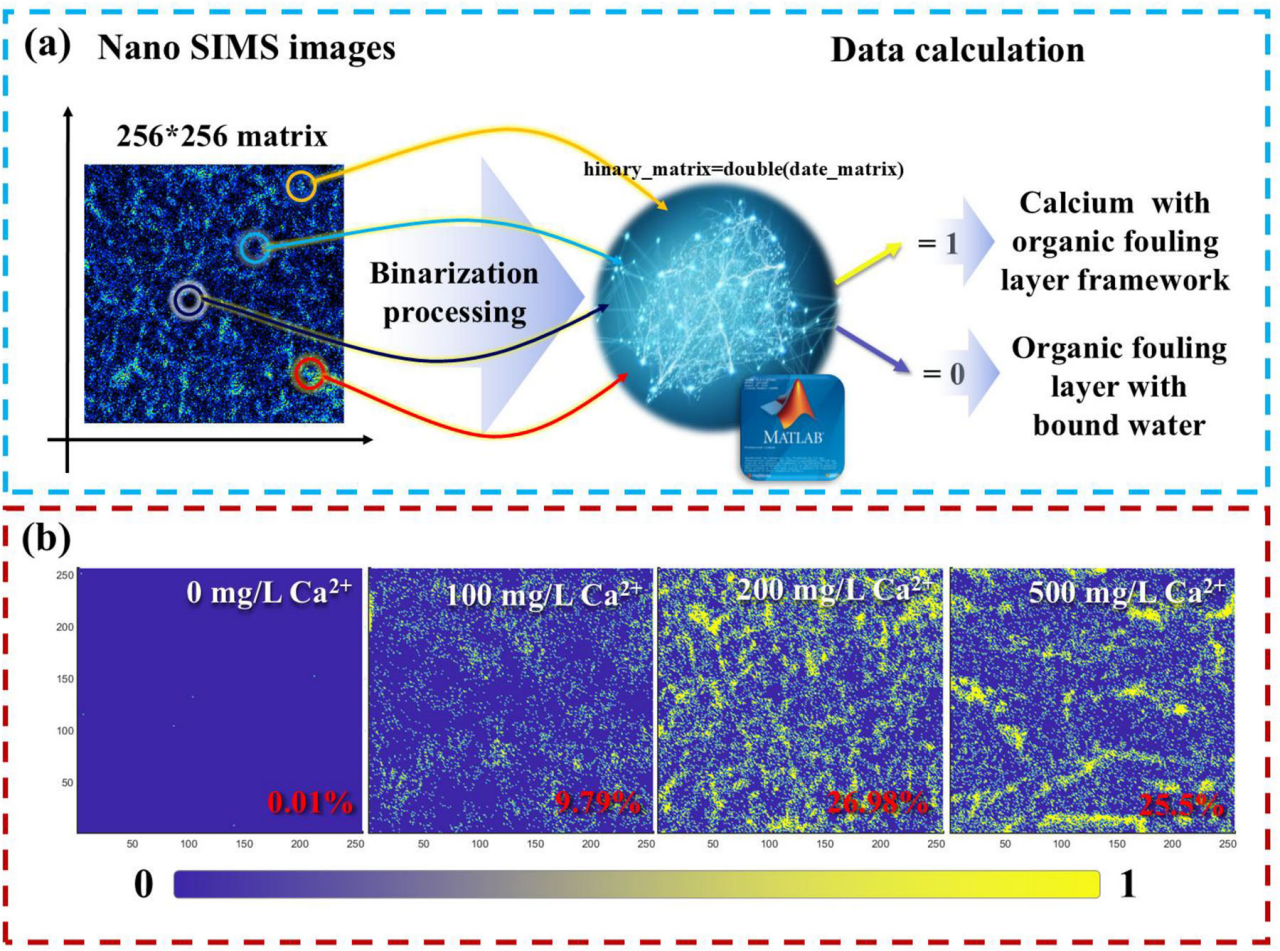
The Nano SIMS images processed through programmed binary thresholding in different Ca^2+^ concentrations. (a) schematic diagram of binarization processing; (b) the result figures.

It is important to note that, in addition to observing the distribution of Ca^2^^+^ in the fouling layer from a comprehensive perspective, this study introduced a novel approach for quantifying its elemental content. As previously described, Nano SIMS uses a Cs^+^ ion source to emit primary ions onto the sample, yielding ionic responses from ^1^ ^2^C^−^, ^1^ ^6^O^−^, and ^4^ ^0^Ca^1^ ^6^O^−^. These responses produce a 256 × 256 matrix of ionic element strength data within a 10 × 10 µm area. To examine the distribution of Ca^2^^+^ within the organic fouling layer, the ion strength matrix generated by Nano SIMS was binarized, as shown in Figure 4a. This process involved selecting the median value of the ^4^ ^0^Ca^1^ ^6^O^−^ matrix and applying it in a binarization algorithm. Data points above the median were assigned a value of “1,” indicating the presence of molecular chains containing Ca^2^^+^ within the organic fouling framework. Conversely, data points below the median were assigned a value of “0,” representing the pores in the fouling layer, which were previously occupied by bound water before drying. Following binarization, the quantification of Ca^2^^+^‐associated organic fouling was achieved by calculating the percentage of “1” values within the entire matrix. The resulting binary data was visualized in Figure 4b. After binarization, the distribution of Ca^2^^+^ in the organic fouling layer became clearer, with the molecular chains containing Ca^2^^+^ accounting for < 0.1%, 9.79 ± 0.12%, 26.98 ± 0.46%, and 25.50 ± 0.21% of the fouling layer, respectively, as the Ca^2^^+^ concentration increased. This increasing trend aligns with previous studies [6, 47], providing concrete evidence that a Ca^2^^+^ concentration of 500 mg/L surpasses the binding threshold for 200 mg/L SA molecules in solution. An excessive concentration of Ca^2^^+^ leads to the aggregation of molecular chains, which reduces the cross‐linking between organic molecules.

These findings underscore the significant contributions of Nano SIMS to the study of membrane fouling. First, Nano SIMS facilitates “visualization” by enabling direct observation of the non‐destructive characterization morphology of the organic fouling layer enhanced by Ca^2^^+^, with clear images of Ca^2^^+^ distribution. Second, the “quantification” aspect is achieved through binarized Nano SIMS results, which demonstrate that the proportion of polymeric compounds increases with the rise in Ca^2^^+^ concentration, thus quantitatively describing the degree of Ca^2^^+^‐induced enhancement of organic fouling.

### 3.4 Nano SIMS Assisted Thermodynamic Understanding of Fouling Mechanisms

**FIGURE 5:**
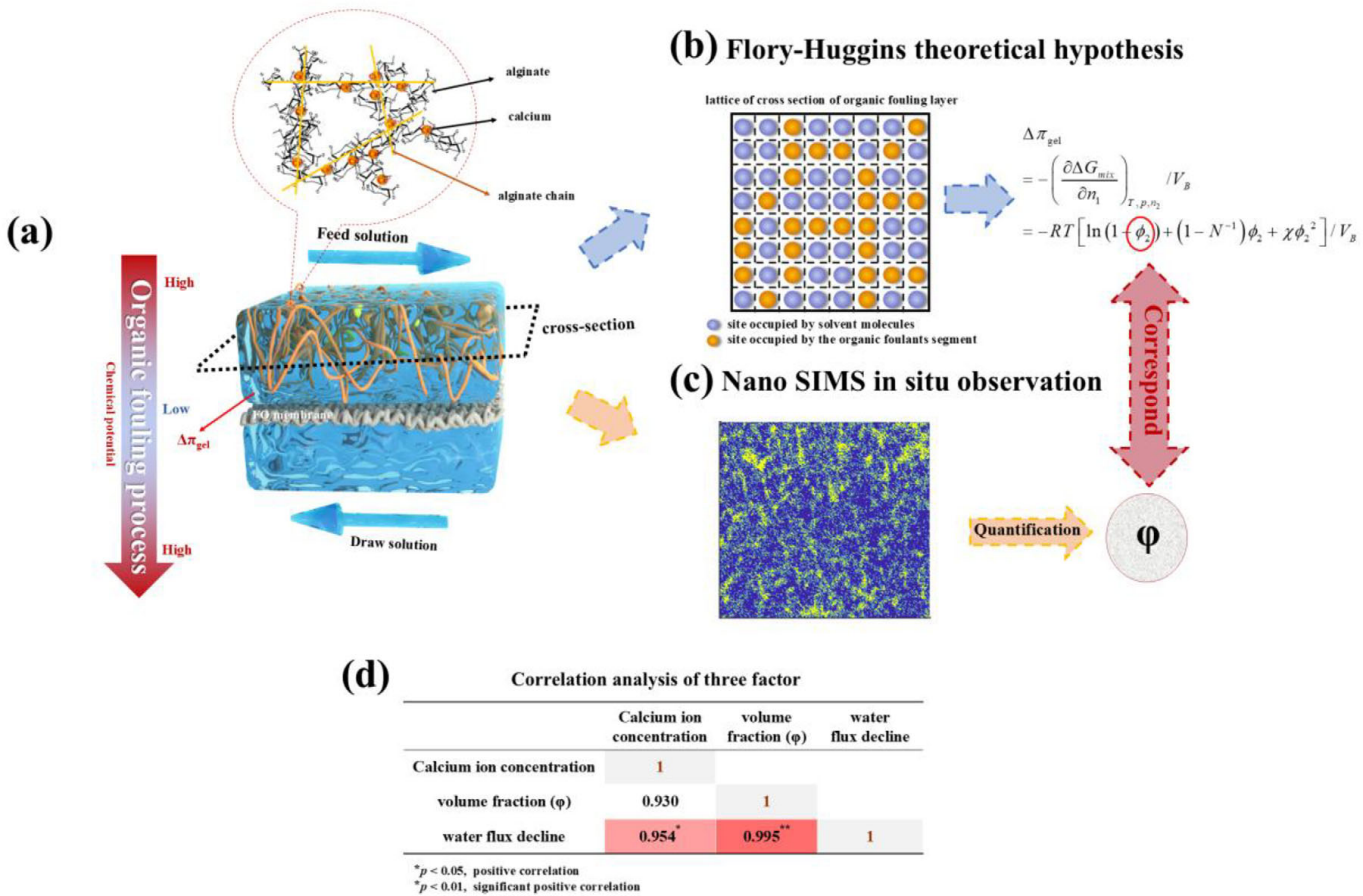
Schematic diagram of (a) the cross‐linking process of SA with calcium ions and the organic layer filtration process in the FO system; (b) Flory–Huggins theoretical hypothesis corresponding to (c) the Nano SIMS images; (d) correlation analysis of three factor.

Flory‐Huggins theory introduces the concept of chemical potential change (Δµ_m_ *_ix_*) to explain organic fouling mechanisms [48, 49]. Further details are provided in the Text S2. Although the theory's validity has been confirmed by numerous studies, some parameters remain challenging to determine accurately. Specifically, macromolecular polymers (represented by SA molecules and Ca^2^^+^) in the cross‐section of the organic fouling layer are modeled as multiple lattice units (as depicted in Figure 5a,b). In this model, the yellow points correspond to positions occupied by segments of organic foulants (SA chains), while the blue points represent solvent molecules (typically bound water). Based on this assumption, the volume fraction of the SA chain is denoted as *φ_2_*. From the Flory‐Huggins theory perspective, both *φ_2_* and *χ* are crucial factors determining the osmotic pressure (*Δπ_gel_*). The value of *χ* represents the interaction energy between different polymers and is typically derived from empirical data [48, 50], while *φ_2_* is determined by experimental conditions. Although some studies have proposed methods to estimate *φ_2_*, substantial evidence to support these assumptions remains limited [33, 47, 49].

To address these challenges, this study proposes a novel approach based on Nano SIMS results (as shown in Figure 5c). We introduced a method in which the organic polymer content, quantified from binarized Nano SIMS images, is correlated with *φ_2_* in the organic fouling layer. First, Nano SIMS provides data on the content of polymeric compounds in the organic fouling layer at various Ca^2^^+^ concentrations. The binarized Nano SIMS images (Figure 4) were then used to quantify *φ_2_*. The calculated values for *φ_2_* were 0.01, 0.06, 0.17, and 0.16, which closely aligned with the trends of water flux decline shown in Figure 1. This suggests that *φ_2_* can be quantified through the Nano SIMS binarization data. To validate the reliability of this quantification, Pearson correlation analysis was conducted to assess the relationship between water flux, *φ_2_*, and Ca^2^^+^ concentration. The analysis revealed a highly significant positive correlation between *φ_2_* and water flux decline (^**^ *p* < 0.01), followed by a correlation between Ca^2^^+^ concentration and water flux decline (*p < 0.05) (Figure 5d). The consistency between the water flux data and *φ_2_* predictions supports the feasibility of the proposed Nano SIMS method. Mechanistically, the increase in *φ_2_* from 0.01 to 0.17 reflects the progressive densification of the Ca‐SA gel network as Ca^2^^+^ concentration increases from 0 to 200 mg/L. These findings indicate that non‐destructive characterization observations of the Ca^2^^+^‐enhanced organic layer using Nano SIMS can enhance our understanding of the thermodynamic model described by the Flory‐Huggins theory in the context of severe membrane fouling. Furthermore, the results from this study offer a new perspective with broad applicability in addressing membrane fouling challenges across various membrane technologies.

### 3.5 Non‐destructive Characterization Analysis of Membrane Fouling Induced by Real Landfill Leachate Using Nano SIMS

**FIGURE 6:**
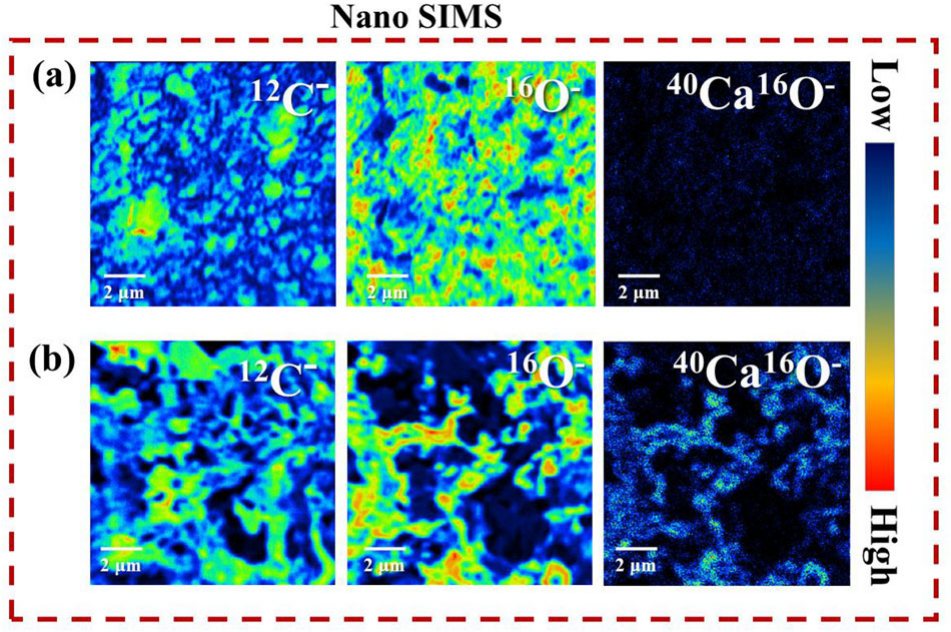
Nano SIMS images of the fouled FO membranes with the real landfill leachate as FS. (a) pH of 4.5; (b) pH of 7.0.

To enhance the environmental relevance of this study, real landfill leachate with a complex matrix of organic matter and multiple ions was selected for analysis (detailed water quality and metal content are provided in Tables S4 and S5). SEM and EDX results (Figure S2) revealed that after 12 h of filtration, the Ca^2^^+^ content in the FO membrane fouling layer was 3.5% at pH 4.5 and 13.8% at pH 7.0 (Table S6). Nano SIMS analysis (Figure 6) further illustrated the fouling behavior of landfill leachate on FO membranes. The strong signals of ^1^ ^2^C^−^ and ^1^ ^6^O^−^ in both fouling layers confirmed the abundant presence of organic foulants on the membrane surface, which can be attributed to the high organic load of the leachate. However, the markedly higher ^4^ ^0^Ca^1^ ^6^O^−^ signal at pH 7 indicated more significant calcium deposition, consistent with the EDX findings in Table S6. These results align with previous studies showing that under acidic conditions, organic foulants exhibit minimal complexation with Ca^2^^+^, thereby reducing calcium‐associated fouling [42]. As shown in Table S6, the landfill leachate contained additional contaminants (e.g., sulfate, barium), yet these did not interfere with Nano SIMS measurements. This reflects the strong ion‐recognition specificity of the technique, confirming its robustness for analyzing membrane fouling in complex matrices.

### 3.6 Limitations and Future Perspectives of Nano SIMS

While Nano SIMS offers valuable insights into the chemical composition of the fouling layer, several limitations should be considered. First, although Nano SIMS achieves high spatial resolution (100–200 nm), this scale remains several orders of magnitude larger than the molecular dimensions of Ca‐SA crosslinked networks (2–5 nm). As a result, the observed network‐like patterns do not directly resolve individual Ca^2^^+^‐SA molecular crosslinks. Second, reported *φ_2_* values represent the spatial coverage of Ca^2^^+^‐bound SA networks at the Nano SIMS resolution (100–200 nm), rather than true molecular‐scale stoichiometric ratios of Ca^2^^+^ to SA. The binarization approach is applied solely to the Nano SIMS images to facilitate clear visualization. And third, all characterizations were performed ex situ after filtration. Despite careful sample preservation using cryogenic freezing and freeze‐drying, some structural reorganization during sample preparation cannot be entirely excluded.

Despite these limitations, Nano SIMS provides a significant advancement in mesoscale characterization of membrane fouling, offering a critical link between molecular‐level interactions and macroscopic membrane performance. Future research could focus on combining Nano SIMS with elemental mapping of inorganic ions (e.g., Ca, Mg, and Al) to track specific molecular species in membrane fouling, and extending this approach to other foulants such as proteins, humic substances, and inorganic colloids across different membrane types.

## 4 Conclusions

This study demonstrates that Nano SIMS was employed for the first time to observe the enhancement of Ca^2^^+^ on organic fouling. It provided non‐destructive visualization of the distribution and intensity of organic foulants, indicating that higher Ca^2^^+^ concentrations worsened organic fouling, consistent with traditional non‐destructive detection methods like SEM, EDX, and CLSM. Additionally, it revealed the spatial distribution and content of Ca^2^^+^ within the fouling layer, demonstrating that 200 mg/L Ca^2^^+^ concentration had the most significant complex‐strengthening effect on the organic foulants. Through binarization of the Nano SIMS image data, the amount of Ca^2^^+^ associated with organic polymers *(φ_2_*) was quantified, showing values of 0.01, 0.06, 0.17, and 0.16 as the Ca^2^^+^ concentration increased. These findings offer strong evidence supporting the Flory–Huggins theory to explain membrane fouling behavior. Nano SIMS provides a unique perspective for visualizing the true state of the organic fouling layer on membranes non‐destructively and is expected to become a powerful tool for investigating organic fouling in membrane processes, facilitating non‐destructive characterization analysis of complex fouling mechanisms.

## Conflicts of Interest

The authors declare no conflict of interest.

## Supporting information




**Supporting File**: advs74734‐sup‐0001‐SuppMat.doc.

## Data Availability

The data that support the findings of this study are available from the corresponding author upon reasonable request.
