# Supplementary material for: Nanoscale Secondary Ion Mass Spectrometry for Quantifying Membrane Fouling: Fouling Layer Structure and Chemical Interaction
Source: Adv Sci (Weinh). 2026 Mar 9:e74734. Online ahead of print. doi: 10.1002/advs.74734 (PMC13325637; doi:10.1002/advs.74734)
Supplement: Supplementary file 1 — Supporting File: advs74734‐sup‐0001‐SuppMat.doc. [file ADVS-9999-e74734-s001.doc]

**Cover Page for Supporting Information**

**Manuscript title:**

Nanoscale Secondary Ion Mass Spectrometry for Quantifying Membrane Fouling: Fouling Layer Structure and Chemical Interaction

**Author list:**

Mengfei Wu^a^, Xinhua Wang*^a,b^, Pin Zhao^a^, Weilong Song^a^, Ming Xie*^c^

^a^ Jiangsu Key Laboratory of Anaerobic Biotechnology, School of Environment and Ecology, Jiangnan University, Wuxi 214122, PR China

^b^ Jiangsu Collaborative Innovation Center of Technology and Material of Water Treatment, Suzhou University of Science and Technology, Suzhou 215009, PR China

^c^ Department of Chemical Engineering, University of Bath, Bath, BA2 7AY, UK

**Total number of pages (including the cover page): 14 pages**

Page 3: Text 1 Comparison of Different Analytical Techniques.

Page 4: Text 2 Flory-Huggins Theory.

Page 4: Table S1 Comparison of characterization techniques for membrane fouling analysis.

Page 6: Table S2 The EDX analysis of element composition on the FO membranes fouled by synthetic wastewater.

Page 7: Table S3 Structural parameters of FO membrane fouling layer from CLSM images fouled by synthetic wastewater.

Page 8: Table S4 The water quality of the real landfill leachate.

Page 9: Table S5 The analysis of element composition of the real landfill leachate.

Page 10: Table S6 The EDX analysis of element composition on the FO membranes fouled by real landfill leachate.

Page 11: Fig. S1. Schematic of the FO setup.

Page 12: Fig. S2. The SEM and EDX images of fouled FO membranes at different landfill leachates. (a) pH of 4.5; (b) pH of 7.

**Text 1 Comparison of Different Analytical Techniques.**

Nano-SIMS uniquely combines high spatial resolution (50-200 nm), quantitative elemental analysis, and multi-element mapping capabilities. It is particularly well-suited for studying sub-micrometer spatial distributions of specific elements (e.g., Ca²⁺, Fe³⁺) in fouling layers where quantitative chemical information is required. However, it should be used in combination with complementary techniques (CLSM for hydrated structure, SEM for morphology, Raman for molecular identity) for comprehensive fouling characterization. At present, numerous studies have conducted extensive exploration of analytical techniques. In order to distinguish the uniqueness of Nano SIMS technology in the field of membrane analysis, we have summarized the following comparisons:

**Table S1** Comparison of characterization techniques for membrane fouling analysis.

| Technique | Spatial Resolution | Elemental  Sensitivity | Quantitative | Sample Requirement | Key Advantages | Key Limitations | Typical Application in Fouling Studies |
| --- | --- | --- | --- | --- | --- | --- | --- |
| Nano SIMS | 50-200 nm | ppm-ppb | Yes (with standards) | Vacuum-compatible, conductive coating | -Highest spatial resolution for chemical mapping  -Multi-element simultaneous detection  -Isotopic discrimination  -Depth profiling | -Destructive (sputtering)  -Ex-situ only  -Expensive, specialized  -Small field of view | -Quantitative elemental mapping,  -isotope tracing,  -chemical speciation |
| ToF SIMS | 1-5 μm | ppb | Semi-quantitative | Vacuum-compatible | - Wide mass range  - Molecular identification  - 3D imaging possible | -Lower spatial resolution  -Matrix effects  -Challenging quantification | -Molecular fingerprinting,  -organic contaminant identification |
| SEM EDX | 1-2 μm | 0.1-1 wt% | Semi-quantitative | Vacuum-compatible, conductive | -Widely available  -Morphology + composition  -Fast analysis | -Poor sensitivity for light elements  -Sample drying required  -Large interaction volume | -Morphology observation,  -elemental composition screening |
| CLSM | 200-500 nm (lateral)  500-800 nm (axial) | N/A (fluorescence  -based) | Semi-quantitative (intensity) | Transparent, hydrated  sample | -Hydrated state imaging  -Non-destructive  -3D reconstruction  -Live-dead staining | -Requires labeling/staining  -Photobleaching  -Limited chemical information  -Semi-quantitative | -Biofilm structure  -fouling layer thickness  -live/dead bacteria |
| AFM | <10 nm | N/A | No (topography) | Flat,  stable surface | -Ultra-high resolution  -Force measurements  -No vacuum needed | -Surface-only  -No chemical information  - Slow for large areas | -Surface roughness  -nanoscale morphology  -adhesion forces |
| XPS | ~10 μm | 0.1 at% | Yes | Vacuum  -compatible | -Surface chemistry  -Chemical state analysis  -Quantitative | -Low spatial resolution  -Surface-sensitive  -Ex-situ | -Surface composition  -oxidation states  -functional groups |
| Raman/FTIR microscopy | 0.5-1 μm | N/A (molecular) | Semi-quantitative | Varies  (can be hydrated  for some modes) | - Molecular identification  -Non-destructive  -Can work in aqueous | -Fluorescence interference  -Weak signals for thin layers  -Limited elemental info | -Functional group mapping  -polymer identification  -EPS characterization |

**Text 2 Flory-Huggins** **Theory.**

The Flory-Huggins theory is a classic thermodynamic model commonly used to describe the thermodynamic changes during polymer mixing processes. Flory-Huggins theory reasonably describes the change of water chemical potential (*Δμ_mix_*) during the formation of fouling layer. According to the principle of Flory-Huggins theory, the filtration process is the transfer of the bound water from the fouling layer (low *Δμ_mix_*) to the permeable liquid (high *Δμ_mix_*), and finally into free water. Therefore, the whole filtration process needs to overcome a large *Δμ_mix_* difference. . Under this assumption, the mixing free energy in a polymer solution can be expressed as:

(S1)

Where *T* is the absolute temperature (K); *R* is the universal gas constant (J·K⁻¹·mol⁻¹); *φ_1_* and *φ_2_* are the volume fractions of the solvent and polymer, respectively; *φ_1_ +φ_2_=1*; *n_1_* and *n_2_* represent the molar amounts of solvent and polymer, respectively; and *χ* is the Flory-Huggins interaction parameter. The change in *Δμ_mix_* during the mixing process of the polymer in the solution can be derived from the partial differential of the Gibbs free energy (*ΔG_mix_*):

(S2)

where *N* is the degree of polymerization, which is typically assumed to be infinite. $\Delta\mu_{mix}=-V_{B}\Delta\pi_{\text{gel}}$ (S3)

where *V_B_* (m^3^·mol^-1^) is the solvent molar volume and the osmotic pressure (*Δπ_gel_*) generated by the organic fouling layer are also considered in the system.

Previous studies indicate that *Δμ_mix_* between the organic fouling layer and the permeate solution is responsible for the additional *Δπ_gel_*, the relationship between Eq. (S1) and (S2) can be expressed as follows:

(S4)

**Table S2** The EDX analysis of element composition on the FO membranes fouled by synthetic wastewater.

|  | **Element** | **Weight %** | **Atomic %** |
| --- | --- | --- | --- |
| SA + 0 mg/L Ca^2+^ | C | 29.7 | 39.8 |
|  | O | 41 | 41.2 |
|  | Na | 23.3 | 16.3 |
|  | Cl | 5.8 | 2.6 |
|  | Ca | 0.2 | 0.1 |
| SA + 100 mg/L Ca^2+^ | C | 3.9 | 7.2 |
|  | O | 27.5 | 37.8 |
|  | Na | 38.2 | 36.5 |
|  | Cl | 27.1 | 16.8 |
|  | Ca | 3.3 | 1.8 |
| SA + 200 mg/L Ca^2+^ | C | 3.8 | 6.5 |
|  | O | 40.7 | 52 |
|  | Na | 32.1 | 28.5 |
|  | Cl | 15.1 | 8.7 |
|  | Ca | 8.3 | 4.3 |
| SA + 500 mg/L Ca^2+^ | C | 11.7 | 18.4 |
|  | O | 44.3 | 52.2 |
|  | Na | 24 | 19.6 |
|  | Cl | 5.9 | 3.1 |
|  | Ca | 14.2 | 6.7 |

**Table S3** Structural parameters of FO membrane fouling layer from CLSM images fouled by synthetic wastewater.

| **Fouling layer** | **Total biovolume (μm^3^)** | **Mean thickness (μm)** |
| --- | --- | --- |
| SA + 0 mg/L Ca^2+^ | 11.75×10^6^±0.12×10^4^ | 26.09±1.34 |
| SA + 100 mg/L Ca^2+^ | 12.99×10^6^±0.53×10^4^ | 36.15±2.67 |
| SA + 200 mg/L Ca^2+^ | 16.24×10^6^±1.58×10^4^ | 64.41±4.84 |
| SA + 500 mg/L Ca^2+^ | 18.24×10^6^±1.99×10^4^ | 67.21±4.62 |

**Table S4** The water quality of the real landfill leachatea.

| **Water quality index** | **Concentration (mg/L)** |
| --- | --- |
| COD | 4877±63.9 |
| TP | 10.44±0.72 |
| TN | 502.7±2.09 |
| NH_4_^+^-N | 504.3±1.87 |

^a^ Values are given as mean values ± standard deviation (number of measurements: n = 3).

**Table S5** The analysis of element composition of the real landfill leachate.

| **Element** | **Concentration** |
| --- | --- |
| Carbonate (mg/L) | 390.1 |
| Sulfate (mg/L) | 282.5 |
| Chloride ions (mg/L) | 2419 |
| Silicate (mg/L) | 9.28 |
| Ca (mg/L) | 125.1 |
| Mg (mg/L) | 24.7 |
| Na (mg/L) | 1898 |
| K (mg/L) | 229.2 |
| Zn (ug/L) | 31.27 |
| Cu (ug/L) | 9.89 |
| Ba (ug/L) | 92.70 |

**Table S6** The EDX analysis of element composition on the FO membranes fouled by real landfill leachate.

| **The landfill leachate** | **Element** | **Weight (%)** |
| --- | --- | --- |
| pH=4.5 | C | 43.8 |
|  | N | 6.8 |
|  | O | 29.2 |
|  | F | 2.5 |
|  | Na | 2.2 |
|  | Mg | 0.2 |
|  | Al | 0.4 |
|  | Si | 0.3 |
|  | P | 6.8 |
|  | S | 0.6 |
|  | Cl | 2.6 |
|  | K | 1.1 |
|  | Ca | 3.5 |
|  | Ba | 0.2 |
| pH=7.0 | C | 40.0 |
|  | N | 11.7 |
|  | O | 25.2 |
|  | F | 0.9 |
|  | Na | 1.2 |
|  | Mg | 0.5 |
|  | Al | 0.4 |
|  | Si | 0.5 |
|  | P | 4.2 |
|  | S | 1.4 |
|  | Cl | 0.1 |
|  | K | 0.0 |
|  | Ca | 13.8 |
|  | Ba | 0.2 |


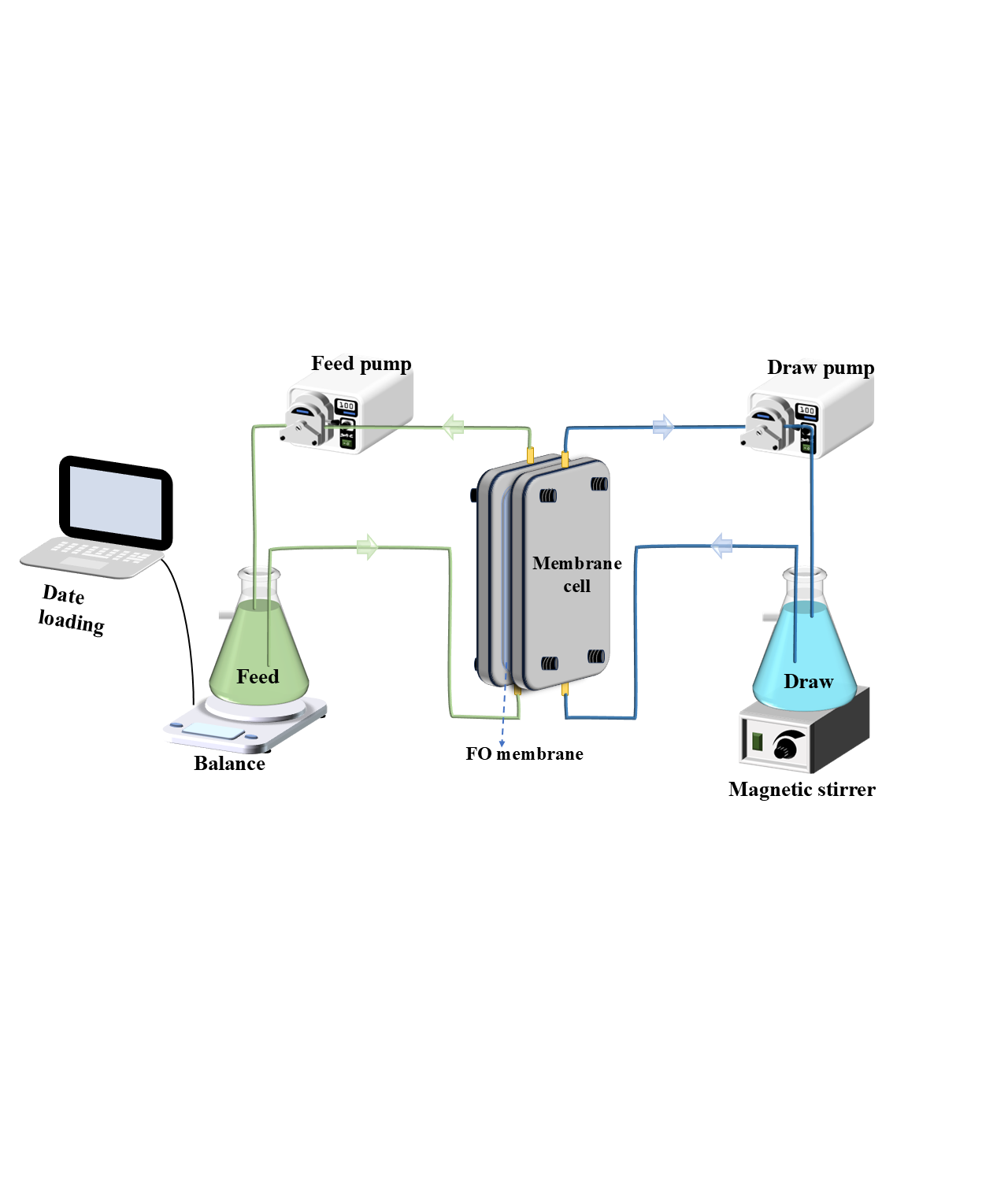


**Fig. S1.** Schematic of the FO setup.


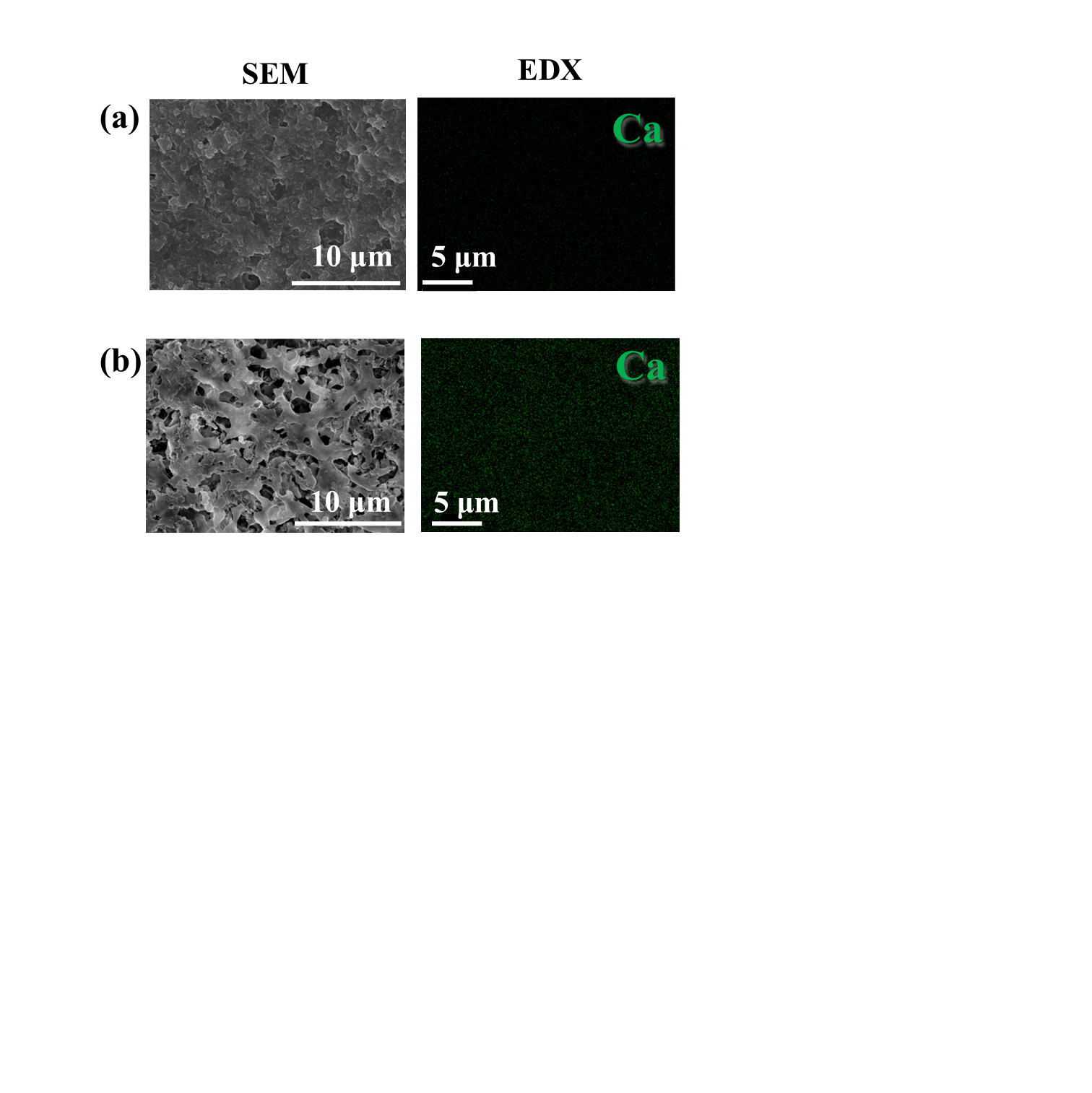


**Fig. S2.** The SEM and EDX images of fouled FO membranes at different landfill leachates. (a) pH of 4.5; (b) pH of 7

# References

Alsteens, D., Gaub, H.E., Newton, R., Pfreundschuh, M., Gerber, C., Müller, D.J., 2017. Atomic force microscopy-based characterization and design of biointerfaces. Nat. Rev. Mater. 2(5), 1-16.

Chen, H., Xia, A., Yan, H., Huang, Y., Zhu, X., Zhu, X., Liao, Q., 2024. Mass transfer in heterogeneous biofilms: Key issues in biofilm reactors and AI-driven performance prediction. Environ. Sci. Ecotechnol., 100480.

Chen, J., Zhang, M., Li, F., Qian, L., Lin, H., Yang, L., Wu, X., Zhou, X., He, Y., Liao, B.-Q., 2016. Membrane fouling in a membrane bioreactor: High filtration resistance of gel layer and its underlying mechanism. Water Res. 102, 82-89.

Flory, P.J., 1942. Thermodynamics of high polymer solutions. J. Chem. Phys. 10(1), 51-61.

Guo, J., Hu, Y., Qian, Y., Shi, Y., An, D., 2024. Review analysis and challenges of sludge-conditioner interactions: Promoting the harmonious integration of experiments and simulations. Chem. Eng. J. 496, 153983.

Hao, L., Liss, S.N., Liao, B.Q., 2016. Influence of COD:N ratio on sludge properties and their role in membrane fouling of a submerged membrane bioreactor. Water Res. 89, 132-141.

Huggins, M.L., 1942. Some properties of solutions of long-chain compounds. J. Phys. Chem. C 46(1), 151-158.

Li, Q., Chang, J., Li, L., Lin, X., Li, Y., 2023. Research progress of nano-scale secondary ion mass spectrometry (NanoSIMS) in soil science: Evolution, applications, and challenges. Sci. Total Environ., 167257.

Sun, C., Lux, S., Müller, E., Meffert, M., Gerthsen, D., 2020. Versatile application of a modern scanning electron microscope for materials characterization. J. of Mater. Sci. 55(28), 13824-13835.

Wu, S., Hua, X., Ma, B., Fan, H., Miao, R., Ulbricht, M., Hu, C., Qu, J., 2021. Three-Dimensional Analysis of the Natural-Organic-Matter Distribution in the Cake Layer to Precisely Reveal Ultrafiltration Fouling Mechanisms. Environ. Sci. Technol. 55(8), 5442-5452.

Yu, G., Xiao, J., Hu, S., Polizzotto, M.L., Zhao, F., McGrath, S.P., Li, H., Ran, W., Shen, Q., 2017. Mineral availability as a key regulator of soil carbon storage. Environ. Sci. Technol. 51(9), 4960-4969.

Yuan, B., Wang, X., Tang, C., Li, X., Yu, G., 2015. In situ observation of the growth of biofouling layer in osmotic membrane bioreactors by multiple fluorescence labeling and confocal laser scanning microscopy. Water Res. 75, 188-200.
